# Supplementary material for: A dosimetric and robustness analysis of proton arc therapy with early energy layer and spot assignment for lung cancer versus conventional intensity modulated proton therapy
Source: Acta Oncol. 2024 Oct 29;63:40549. doi: 10.2340/1651-226X.2024.40549 (PMC11538483; doi:10.2340/1651-226X.2024.40549)
Supplement: A dosimetric and robustness analysis of proton arc therapy with early energy layer and spot assignment for lung cancer versus conventional intensity modulated proton therapy [file AO-63-40549-s1.pdf]

Supplementary material has been published as submitted. It has not been copyedited, or typeset by Acta Oncologica

Supplementary material SM1. Tumor characteristics for the dataset of 14 lung cancer patients.

Patient	Location	Nodal involvement	CTV tumor size on pCT [cm3]	Tumor motion amplitude [mm]		
				R-L	A-P	S-I
1	RML	N	152.6	4.2	2.1	3.1
2	LUL	Y	165.1	1.1	1.1	2.0
3	RML	Y	331.2	0.4	0.5	1.9
4	RUL	Y	146.4	1.2	0.7	1.6
5	RUL	Y	343.4	0.4	1.4	0.5
6	LLL	Y	177.6	3.5	3.0	3.4
7	RUL	Y	79.1	1.3	3.5	0.7
8	RUL	Y	133.7	0.9	0.5	2.9
9	LUL	Y	442.5	0.7	0.8	0.3
10	LUL	Y	130.2	0.8	0.9	1.5
11	RUL	N	109.6	2.2	1.8	6.6
12	RLL	Y	294.4	2.1	2.5	10.6
13	RML	Y	218.1	1.0	3.2	4.9
14	RUL	Y	268.1	4.3	2.8	3.0

Table SM1: Patient characteristics: tumor location, nodal involvement, CTV size and tumor motion amplitude given in absolute value. Abbreviations: LLL = left lower lobe; LUL = left upper lobe; RLL = right lower lobe; RML = right middle lobe; RUL = right upper lobe; Y = Yes; N= No; pCT = Planning CT; R-L = Right-Left; AP = Anterior-Posterior; SI = Superior-Inferior

Supplementary material SM2. ProteusPlus machine parameters used in the beam delivery time (BDT) calculation model for PAT and IMPT plans.

ELST up [s]	6.0
ELST down [s]	0.8
Gantry max velocity [deg/s]	6.0
Gantry max acceleration [s2]	0.6
Time per spot switch [ms]	2
Dead time per energy layer [s]	0.3

Table SM2. Relevant machine parameters (ProteusPlus proton therapy machine) for BDT calculation.

Supplementary Material SM3. IMPT and PAT plan characteristics for the dataset of 14 lung cancer patients

**Patient**	**IMPT**			**PAT**		
	Beams (B1,B2,B3) (degrees)	*Couch kick*	*Range Shifter*	*(arc start, arc stop) (degrees)*	*#Revolutions*	*Gantry angle spacing (degrees)*
**1**	(190,240,290)	0	N	(310,190) (CCW)	1	2
**2**	(15,90,165)	0	Y	(30,170) (CW)	1	2
**3**	(220,270,310)	0	N	(180,0) (CW)	1	1
**4**	(200,270,340)	0	N	(330,190) (CCW)	1	2
**5**	(325,270,210)	0	N	(180,0) (CW)	1	1
**6**	(110,170,230)	0	N	(50,210) (CW)	1	2
**7**	(190,240,300)	0	N	(340,200) (CCW)	1	2
**8**	(190,240,340)	0	N	(0,180) (CCW)	1	2
**9**	(120,160,200)	0	N	(20,160) (CW)	1	2
**10**	(90,130,170)	0	N	(30,170) (CW)	1	1,5
**11**	(170,210,250)	0	N	(320,190) (CCW)	1	1
**12**		0	N	(180,0) (CW)	1	1
**13**	(170,270,220)	0	N	(190,300) (CW)	1	1
**14**	(180,225,270)	0	N	(180,0) (CW)	1	1

Table SM3. Plan characteristics: beam angles (IMPT), couch rotation, presence of a range shifter, arc range (PAT), number of revolutions around the patient and gantry angle spacing. Abbreviations: B1,B2,B3 = beam 1, 2 and 3, CW = clockwise, CCW = counterclockwise.

Supplementary material SM4. CTV Margin calculation for robust optimization

Maximum setup errors were calculated using systematic (Σ) and random (σ) setup and baseline shift values were used for two cases: tumor only and tumor with lymph nodes. This was done using van Herk's margin formula (1) with the goal of obtaining a margin that ensures a minimum dose is delivered to 90% of the patient population:

$$M_{PTV} = 2.5\Sigma_{total} + 0.7\sigma_{total} \quad (1)$$

	$X_{(sagittal)}$ (mm)	$Y_{(coronal)}$ (mm)	$Z_{(transverse)}$ (mm)
Σ_{BL}	1.8	1.6	1.9
Σ_{S}	1.6	2	2.4
σ_{BL}	1.6	1.6	2.1
σ_{S}	1.8	2.1	2.1
Σ_{total}	2.41	2.56	3.06
σ_{total}	2.41	2.64	2.97
M_{PTV}	7.71	8.25	9.73
Expanded CTV margin	2.71	3.25	4.73

Table SM4.1 Setup errors for the case with only primary tumor

	$X_{(sagittal)}$ (mm)	$Y_{(coronal)}$ (mm)	$Z_{(transverse)}$ (mm)
Σ_{BL}	1.9	1.6	1.9
Σ_{S}	1.6	2	2.4
σ_{BL}	1.7	1.6	2.1
σ_{S}	1.8	2.1	2.1
Σ_{total}	2.48	2.56	3.06
σ_{total}	2.48	2.64	2.97
M_{PTV}	7.94	8.25	9.73
Expanded CTV margin	2.79	3.25	4.73

Table SM4.2 Setup errors for the case with primary tumor and lymph nodes

The calculated total setup error values for each direction were inputted in our treatment planning system (TPS), RayStation. However, using a margin that exceeds 5 mm in RayStation leads to a significant increase in optimization time due to the system automatically generating intermediate errors to ensure robust coverage, which can be an over-conservative approach. As a workaround, instead of optimizing on the CTV using the full setup error, a patient-specific CTV expansion can be generated to account for part of the setup error. From the full value of the margin, 5 mm is subtracted (in each direction) and subsequently used as an isotropic setup error value. The CTV is then expanded by the remaining amount. For instance, for a setup error of 7.71

mm in the sagittal direction, a CTV expansion of 2.71 mm was robustly optimized with a setup error of 5 mm (7.71 mm = 2.71 mm in the margin + 5 mm in the robust optimization).
The complete setup margin values can be found in the Appendix (Table SM3.1 and Table SM3.2). Optimization was done on the expanded CTV volume to reduce computation time.

The total number of optimization scenarios was 84: 7 (setup errors: ± 5 mm in x,y,z directions, additionally the nominal scenario) × 3 (image conversion errors: ±3%, 0%) × 4 (breathing phases: MidP, maximum inhale, maximum exhale and an additional mid-ventilation phase).

Supplementary Material SM5. Target homogeneity index (HI), conformity index (CI) and body integral dose (ID) definitions for IMPT and PAT plan dosimetry assessment

Target dose homogeneity was evaluated with the Homogeneity index (HI), defined as a slight variation of the formula in [1]

$$HI = \frac{(D1\% - D98\%)}{D\ prescribed}.$$

Target conformity was assessed as well, by means of the Conformity index (CI), defined as [2]

$$CI = \frac{V95\%}{V_T},$$

where $V95\%$ is the volume covered by 95% of the prescribed dose, and V_T is the total volume of the target. The body integral dose was assessed as well, defined as

$$ID = \underline{D}.V,$$

where $\underline{D}$ is the mean dose to the body in Gy, while V is the patient volume in cc.

Supplementary material SM6. Dutch Normal Tissue Complication Probability (NTCP) model and patient characteristics for the dataset of 14 lung cancer patients.

$$NTCP_x = 1/(1 + e^{-S(x)})$$

S(Grade ≥2 pneumonitis) = −4.12 + 0.138 ∗ MeanLungDose − 0.3711 ∗ (Smoking: stopped) − 0.478 ∗ (Smoking: active) + 0.8198 ∗ (Pulmonary comorbidity) + 0.6259 ∗ (Tumor location) + 0.5068 ∗ Age + 0.47 ∗ (Sequential chemotherapy)

S(Grade ≥2 acute esophageal toxicity) = -3.634 + 1.496*ln(MeanEsophagealDose) - 0.0297*(Interval start-stop RT)

S(2 year mortality) = −1.3409 + 0.0590 ∗ $SQRT$(GTVvolume) +0.2635 ∗ SQRT(MeanHeartDose)

	**Dysphagia Gr2**	**Pneumonitis**					
		**Smoking**					
**Patient**	**Overall treatment time (days)**	**Stopped Smoking ***	**Active smoking ****	**Pulmonary Comorbidity*****	**Lobe******	**Seq. Chemotherapy *******	**Age**
1	33	1	0	0	1	0	70
2	41	1	0	1	0	0	79
3	40	1	0	1	1	0	64
4	40	0	1	0	0	0	57
5	44	0	1	1	0	0	62
6	39	1	0	1	1	0	74
7	42	0	0	0	0	0	71
8	40	1	0	0	0	0	79
9	40	0	1	1	0	0	58
10	40	1	0	0	0	0	78
11	40	1	0	0	0	0	70
12	40	0	1	1	1	0	71
13	40	1	0	1	1	0	73
14	40	1	0	1	0	0	58

Table SM6. Clinical characteristics of patients considered for the NTCP calculation. * stopped smoking = 1, never or active smoker = 0 ; ** active smoker = 1, never smoked or quit = 0 ; *** COPD or other pre-existing lung disease = 1, None = 0 ; **** Middle/lower lobe = 1, upper lobe = 0 ; ***** Yes = 1 , No = 0.

For more details, see [3].

Supplementary material SM7. Dosimetric and NTCP results on plan comparison between PAT and IMPT, for the dataset of 14 lung cancer patients

	IMPT		PAT		IMPT-PAT			
**Clinical Goal**	**Median (NC)[Gy]**	**Median (WC)[Gy]**	**Median (NC)[Gy]**	**Median (WC)[Gy]**	**Median (NC)[Gy]**	**p-value**	**Median (WC)[Gy]**	**p-value**
CTV D95%	58,99	57,90	58,90	57,71	0,09	0,15	0,07	0,86
CTV D98%	58,77	56,48	58,61	57,00	0,14	0,12	-0,49	0,05
CTV D1%	61,37	62,09	61,50	63,05	-0,09	0,14	-0,99	0,00012
CTV HI	0,04	-	0,05	-	0,00	0,19	-	-
CTV CI	4,36	-	2,62	-		0,00012	-	-
Esophagus D mean	22,79	27,83	23,64	28,04	-0,43	0,19	-0,86	0,27
Esophagus D0.04 cc	-	63,74	-	65,50	1,43	0,0100	-1,75	0,02
Heart D mean	5,99	9,81	7,29	10,84	-1,41	0,00012	-1,70	0,00012
Heart D0.04 cc	-	64,97	-	65,50	1,05	0,00012	-0,58	0,33
Lungs - GTV D mean	14,12	14,85	13,10	15,07	-0,57	0,24	-0,62	0,36
Lungs V30Gy	17,46	20,37	16,04	18,79	1,56	0,0085	1,69	0,025
Spinal Canal D0.04cc	28,97	36,68	31,80	47,57	-0,68	0,952	-3,13	0,14
Body D1cc	62,61	64,16	62,09	64,77	0,50	0,0017	-0,23	0,15
Body ID [Gy.cc]	121,80	-	147,17	-	-10,62	0,00085	-	-

Table SM7a. Median values per treatment technique, and median values for the difference. NC = nominal case, WC = worst case, ID = integral dose

Strategy	NTCP_pneumonitis	NTCP_2y_mortality	NTCP_dysphagia_g2
IMPT	11.3	45.61	44.79
arcPT	13.185	47.745	47.185

Table SM7b. Median NTCP values per treatment technique.

NTCP metric	Median Delta NTCP (IMPT-PAT)	p value
NTCP_2y_mortality	-1.97	0,00012
NTCP_dysphagia_g2	-0.69	0,14
NTCP_pneumonitis	-1.01	0,24

Table SM7c.Median values for the difference in NTCP between the treatment modalities

Supplementary Material SM8. IMPT and PAT plan characteristics influencing BDT results, and delivery time values for the dataset of 14 lung cancer patients

	Number of beams		total EL		ELS up		ELS down		#Spots		BDT(s)	
**Patient**	IMPT	PAT	IMPT	PAT	IMPT	PAT	IMPT	PAT	IMPT	PAT	IMPT	PAT
1*	3	1	97	61	2	5	94	55	14026	11933	190.8	130.7
2*	3	1	120	71	2	5	117	65	22633	10438	209.1	144.3
3	3	1	110	180	2	17	107	163	27826	28745	240.1	366.1
4*	3	1	112	71	2	5	109	65	26955	15817	247.9	155.7
5	3	1	132	181	2	7	129	173	43684	43997	303.1	339.9
6*	3	1	110	81	2	5	107	75	20469	18622	215.2	171.4
7	3	1	82	71	2	9	79	61	7653	9142	157.5	163.1
8*	3	1	110	91	2	7	107	83	20123	19811	228.2	200.1
9*	3	1	100	71	2	4	97	66	25241	19699	214.8	157.3
10	3	1	88	94	2	11	85	82	15527	23132	172.3	227.6
11	3	1	77	131	2	6	74	124	10276	17389	159.6	222.7
12	3	1	117	181	2	24	114	156	33561	32451	278.3	420.9
13*	3	1	98	111	2	5	95	105	18028	21472	210.6	204.1
14	3	1	96	181	2	5	93	175	18912	20969	205.2	281.8

Table SM8. Plans characteristics: number of beams or arcs, total number of energy layers, energy layer switchings, number of spots and beam delivery time results. Abbreviations: IMPT = intensity modulated proton therapy, PAT = proton arc therapy, EL = energy layers, ELSU = energy layer switching up, ELSD = energy layer switching down, BDT = beam delivery time. * indicate that the PAT plan held a smaller BDT value than the IMPT plan.

References

1. Kataria, T., Sharma, K., Subramani, V., Karrthick, K. P. & Bisht, S. S. Homogeneity Index: An objective tool for assessment of conformal radiation treatments. *J. Med. Phys.* **37**, 207–213 (2012).
2. Feuvret, L., Noël, G., Mazeron, J.-J. & Bey, P. Conformity index: a review. *Int. J. Radiat. Oncol. Biol. Phys.* **64**, 333–342 (2006).
3. Landelijk Platform voor Radiotherapie bij Longtumoren (LPRL) & Landelijk Platform Protonentherapie (LPPT). Landelijk Indicatie Protocol Protonen Therapie Longcarcinoom (2019) . Available from: https://nvro.nl/images/documenten/rapporten/LIPP_longen_final_01122019.pdf
